# Ultrawide Bandwidth Electromagnetic Wave Absorbers Composed of Double-Layer Frequency Selective Surfaces with Different Patterns

**DOI:** 10.1038/s41598-018-32181-z

**Published:** 2018-09-17

**Authors:** Tian Liu, Sung-Soo Kim

**Affiliations:** Department of Advanced Materials Engineering, Chungbuk National University, Cheongju, 361-763 Korea

## Abstract

A novel design for an ultra-wide bandwidth and thin microwave absorber is introduced utilizing two frequency selective surfaces (FSSs) with different patterns of resonating frequencies. The circuit parameters, inductance and capacitance, of the three types of FSS (square loop, cross, square patch) were determined using an equivalent circuit and strip wire conductor model. The square loop FSS indicates a low frequency resonance (10 GHz) due to its high inductance and capacitance. On the other hand, the square patch of small inductance reveals a high resonating frequency (36 GHz). By optimizing the combination of the two FSSs, an ultra-wide absorption bandwidth (6.3–40.0 GHz for −10 dB reflection loss) was designed with a small total thickness of 5.5 mm, which is close to the theoretical limit. The free space measurement result with a test sample prepared by the screen printing method was in good agreement with the simulation result and verified the validity of the proposed design method. For these periodic array structures, however, the grating lobes were observed above the high frequency limit, and it needs to be emphasized that the further control of the unit cell periodicity is important, particularly for large oblique incidence angles.

## Introduction

With the increased use of electronics over the broad frequency spectrum from microwaves to millimeter waves, high-performance absorbing and shielding materials are needed to ensure electromagnetic wave control and compatibility. Acquisition of wide bandwidth absorption with a planar layer and a small layer thickness has been a major challenge for commercial and military applications. Unfortunately, it is a difficult task to achieve both bandwidth enhancement and thickness reduction simultaneously because of an inverse proportional relationship among the bandwidth, thickness, and reflectance of the absorbers, which was theoretically explained by Rozanov^1^.

The Jaumann absorber^2–4^ is one example of broad bandwidth absorbers, but it suffers from a much higher thickness compared to the single-layer Salisbury screens^5^. As one of the demonstrating examples, an eight resistive sheet Jaumann absorber satisfies the 20 dB bandwidth requirement of 2.95–35.2 GHz^6^, but the total thickness of the absorber increases to a large value (31.4 mm), more than twice the theoretical limit. Metamaterials have been proposed to achieve an extremely thin absorber, but they exhibited a very narrow bandwidth^7–9^. The use of frequency selective surfaces (FSS)^10–17^ or metasurfaces^18–21^ on a nonmagnetic substrate is one effective approach to widen the bandwidth while reducing thickness. Single-layer absorbing structure synthesized using a resistive square loop FSS obtained remarkable performance (15 dB in the band from 7 GHz to 20 GHz) with an overall thickness of 5 mm only^10^. An experimental result for a 10 dB absorption bandwidth of 5.27–18 GHz was reported with a 4 mm thick absorber using an FSS of crisscross and fractal square patch^14^. A hexagonal pattern of resistive FSS was proposed for a broadband absorber with 10 dB bandwidth of 6.4–24.9 GHz at a thickness of 4 mm^17^.

Further enhancement of absorption bandwidth has been reported by using the multilayers of FSSs or metasurfaces^22–27^. A periodic array of loop-dielectric multilayered structure was proposed with wideband frequency response covering 8.4–21 GHz for 10 dB absorption and a total thickness of 3.65 mm^22^. Multilayered structure of four split ring resonators (SRRs) with different geometries has been proposed to realize a 90% absorptive bandwidth of 10–70 GHz based on numerical simulation^24^. A broadband polarization-independent circuit analogue absorber comprising multi-layer resistive square loop FSS has been presented^25^, which demonstrated the reflectivity below −10 dB in the frequency range from 4.96 GHz to 18.22 GHz with a total thickness of 4.6 mm. A multilayer absorber composed of three layers of square metasurfaces was demonstrated to realize the ultra-wideband absorption more than 90% from 7.0 GHz to 37.4 GHz with a total thickness of 3.8 mm as low as 0.09λ_L_, where λ_L_ is the wavelength at the lowest operating frequency^26^. Table 1 summarizes the important results of existing works on the broad bandwidth absorbers of multilayered FSS structures. In spite of the extensive studies on wide bandwidth, however, problems still remain for not enough bandwidth or the complex structure with too many resistive sheets, which leads to a thick absorber and complexity in fabrication.

**Table 1:** Summary of the important results of existing works on the broad bandwidth absorbers of multilayer FSS structures.

References	10 dB bandwidth frequency range (GHz)	Fractional bandwidth (%)	Absorber thickness (mm)	Number of FSS
^22^	8.4–21.0	86	3.7 (0.10λ_L_)	3
^23^	6.0–19.0	104	4.4 (0.08λ_L_)	4
^22^	10.0–70.0	150	4.5 (0.15λ_L_)	4
^25^	5.0–18.0	114	4.6 (0.08λ_L_)	2
^26^	7.0–37.4	137	3.8 (0.09λ_L_)	3
This work	6.3–40.0	145	5.5 (0.11λ_L_)	2

The fractional bandwidth is given by the ratio of bandwidth to center frequency. λ_L_ is the wavelength at the lowest operating frequency.

In the previous studies^10–14,16^, the design principle of a single-layer FSS absorber was introduced for obtaining the wide bandwidth through impedance matching at two separated frequencies in a broad frequency span, which determines the absorption bandwidth. The center frequency of the absorption band corresponds to the resonance frequency of the FSS, which can be controlled by the FSS geometries, including unit cell periodicity. By combining two arrays of FSSs with different geometries (thus with different resonance frequencies), a significant enlargement of absorption bandwidth was achieved^28^. Extending this idea, an FSS combination with different patterns would be possible for designing the ultra-wide bandwidth absorbers. The resonance frequency can be more efficiently controlled through selecting the appropriate FSS patterns; for example, a square loop FSS is high in inductance and capacitance (thus having low resonating frequency) and a square patch has a low inductive and high-frequency resonating element. The two FSS combination makes it possible to design low-reflectivity absorbers in a broader frequency span while maintaining a small layer thickness.

In this study, a thin and ultra-wide bandwidth microwave absorber was demonstrated utilizing two frequency selective surfaces with different resonating frequencies. Three types of FSS (square loop, cross, square patch) were investigated for the systematic control of the circuit parameters of inductance and capacitance, and thus the resonating frequencies. Through a combination of two FSS patterns, an ultra-wide absorption bandwidth (6.3–40.0 GHz for −10 dB reflection loss) could be designed with a small total thickness of 5.5 mm, which is close to the theoretical limit. That is the novelty of this study over the existing works from standpoints of enlarged bandwidth (fractional bandwidth 145%) with a comparable thickness (0.11λ_L_) and simple structure (only two FSS layers) of easy fabrication, as compared in Table 1. We could state the possible mechanism based on the coupling behavior between the double-layer FSSs. The free space measurement result with a test sample prepared by the screen printing method was in good agreement with the simulation result and strongly verified the validity of the proposed design method. In addition, the angular stability of the proposed wide bandwidth absorber was investigated over wide oblique incidence angles for both TE and TM polarization in association with unit cell periodicity and grating lobes or high-frequency harmonics.

## Results

### Inductance and capacitance of FSSs

**Figure 1:**
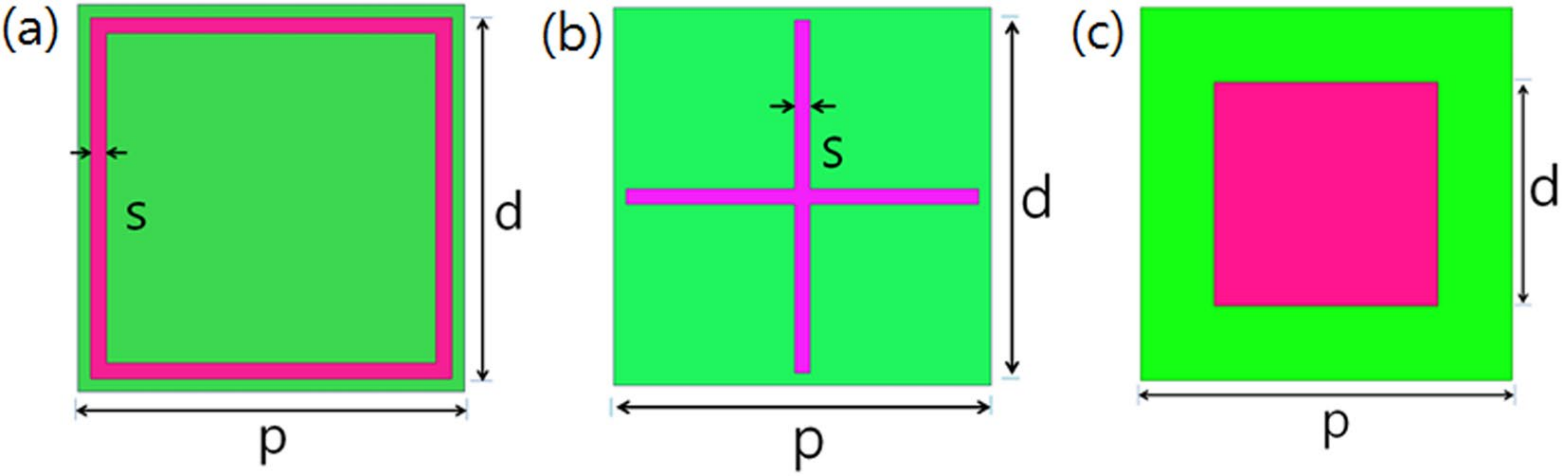
Schematic of the three types of FSSs and their dimensions: (**a**) square loop FSS (p = 7.5 mm, d = 7.0 mm, s = 0.3 mm), (**b**) cross FSS (p = 7.5 mm, d = 7.0 mm, s = 0.3 mm), and (**c**) square patch FSS (p = 7.5 mm, d = 4.5 mm).

Figure 1 illustrates the three types of FSS (square loop, cross, square patch) and their dimensions. The dimensions of the unit cell period (p), FSS length (d), FSS width (s), and interval space (g) between the two FSSs are given in Fig. 1. For the square loop FSS (designated as SL-FSS), p = 7.5 mm, d = 7.0 mm, s = 0.3 mm, and g = 0.5 mm; for the cross FSS (designated as C-FSS), p = 7.5 mm, d = 7.0 mm, s = 0.3 mm, and g = 0.5 mm; for the square patch FSS (designated as SP-FSS), p = 7.5 mm, d = 4.5 mm, and g = 3.0 mm.

**Figure 2:**
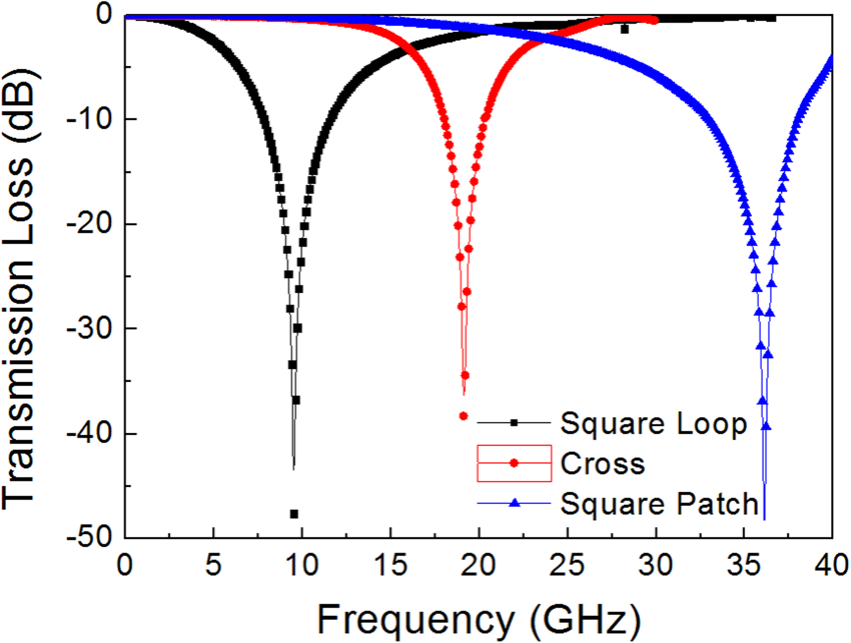
Simulation results of the transmission loss for the three types of FSS (square loop, cross, square patch) standing in free space with the dimensions given in Fig. 1.

Resonance frequency can be determined from the transmission loss for the FSS structures which can be represented as an equivalent circuit of shunted *L-C* series circuit. Figure 2 presents the simulation results that this array had a single principal resonance at a frequency (*f*_0_) of 9.5 GHz for SL-FSS, 19.1 GHz for C-FSS, and 36.1 GHz for SP-FSS. For the SL-FSS and C-FSS, the inductance and capacitance were calculated from the equations of reactance and susceptance at the resonance frequency of the equivalent *L-C* circuit, which have been provided in the literature^16,29,30^. For the SL-FSS and C-FSS with the geometries illustrated in Fig. 1(a) and 1(b), the inductance and capacitance were calculated to be *L* = 2.95 nH and *C* = 83.04 fF for SL-FSS and *L* = 6.23 nH and *C* = 10.62 fF for C-FSS. For the SP-FSS, a strip wire conductor model^31^ was applied to determine *L* and *C* and were calculated to be *L* = 0.74 nH and *C* = 26.50 fF for the dimension shown in Fig. 1(c). Table 2 summarizes the results. With these *L* and *C* values, the resonance frequency was *f*_0_ = 1/[2π(*LC*)^1/2^] = 10.1 GHz for SL-FSS, *f*_0_ = 19.6 GHz for C-FSS, and *f*_0_ = 36.1 GHz for SP-FSS, which is consistent with the simulation results presented in Fig. 2.

**Table 2:** Circuit parameters (*L*, *C*) and resonance frequencies of FSSs.

FSS	*L* [nH]	*C* [fF]	*f*_0_ (calculation)	*f*_0_ (simulation)
Square Loop	2.95	83.04	10.1	9.6
Cross	6.23	10.62	19.6	19.1
Square Patch	0.74	26.50	36.1	36.1

### Single-layer FSS Absorber Design

First we designed a single-layer microwave absorber composed of an FSS on the top layer and a perfect electric conductor (PEC) on the bottom layer. For simplicity, the substrate material was assumed to be air and we can use the *L* and *C* values of the FSS as determined previously. The reflection loss was determined by the total admittance of the high impedance surface (Y_total_), which was equal to the parallel connection between the surface admittance of the grounded dielectric slab (Y_t_) and the complex admittance of FSS (Y_fss_):

1 $${{\rm{Y}}}_{{\rm{t}}{\rm{o}}{\rm{t}}{\rm{a}}{\rm{l}}}={{\rm{Y}}}_{{\rm{t}}}+{{\rm{Y}}}_{{\rm{f}}{\rm{s}}{\rm{s}}}={{\rm{Y}}}_{0}[{\rm{R}}{\rm{e}}({{\rm{Y}}}_{{\rm{t}}{\rm{o}}{\rm{t}}{\rm{a}}{\rm{l}}})+{\rm{j}}{\rm{I}}{\rm{m}}({{\rm{Y}}}_{{\rm{t}}{\rm{o}}{\rm{t}}{\rm{a}}{\rm{l}}})]$$

2 $${{\rm{Y}}}_{{\rm{t}}}=-\,{{\rm{Y}}}_{0}\sqrt{\frac{{\varepsilon }_{r}}{{\mu }_{r}}}\,\coth ({\rm{j}}\frac{2\pi t}{\lambda }\sqrt{{\varepsilon }_{r}{\mu }_{r}})={{\rm{Y}}}_{0}[{\rm{R}}{\rm{e}}({{\rm{Y}}}_{{\rm{t}}})+{\rm{j}}{\rm{I}}{\rm{m}}({{\rm{Y}}}_{{\rm{t}}})]$$

3 $${{\rm{Y}}}_{{\rm{f}}{\rm{s}}{\rm{s}}}=\frac{1}{{\rm{R}}+{\rm{j}}(\omega L-\frac{1}{\omega C})}={{\rm{Y}}}_{0}[{\rm{R}}{\rm{e}}({{\rm{Y}}}_{{\rm{f}}{\rm{s}}{\rm{s}}})+{\rm{j}}{\rm{I}}{\rm{m}}({{\rm{Y}}}_{{\rm{f}}{\rm{s}}{\rm{s}}})]$$

Re(Y_total_) and Im(Y_total_) are the real and imaginary parts of the total admittance of a high impedance surface normalized by the free space admittance (Y_0_ = 1/377 Ω^−1^). Admittance matching condition for zero reflection is given by Re(Y_total_) = 1 and Im(Y_total_) = 0.

The normalized input admittance of a grounded dielectric substrate (Y_t_/Y_0_ = Re(Y_t_) + jIm(Y_t_)) was determined from the relative permittivity (${\varepsilon }_{r}$), relative permeability (${\mu }_{r}$), and thickness (*t*) of the substrate. The short-circuit resonance was found at the frequency where Im(Y_t_) = 0, for which the spacer thickness equals a quarter wavelength ($\lambda /4$ = 7.8 mm, 3.8 mm, 2.0 mm for SL-FSS, C-FSS, SP-FSS, respectively).

The normalized complex admittance of the FSSs (Y_fss_/Y_0_ = Re(Y_fss_) + jIm(Y_fss_)) can be derived from the circuit parameters (*R*, *L*, *C*). The previously determined *L* and *C* values were used, and *R* was controlled by varying the surface resistance of the FSS conductors. The FSS resistance (*R*) had the following relationship with the surface resistance of conductor (*R*_s_): $R\approx {R}_{s}(B/A)$, where *A* is the effective area of current flow and *B* is the unit cell area (= p × p). Table 3 presents the *R* values of the three types of FSSs with an increasing surface resistance. The circuit resistance of FSSs is constant, not dispersive with frequency. At an appropriate surface resistance of FSS, the real part approaches the free-space admittance (Re(Y_fss_) = 1). The resonance frequency, at which Im(Y_fss_) = 0 and calculated from *f*_0_ = 1/[2π(*LC*)^1/2^], is 9.6 GHz for SL-FSS, 19.6 GHz for C-FSS, and 36.1 GHz for SP-FSS, respectively.

**Table 3:** Circuit resistance (*R*) of FSSs with an increasing surface resistance of FSS conductors (*R*_*s*_).

Surface resistance, *R*_*s*_ (Ω/sq)	10	20	100	200
SL-FSS, *R* (Ω)	134	268	1340	2680
C-FSS, *R* (Ω)	268	536	2680	5360
SP-FSS, *R* (Ω)	28	56	280	560

**Figure 3:**
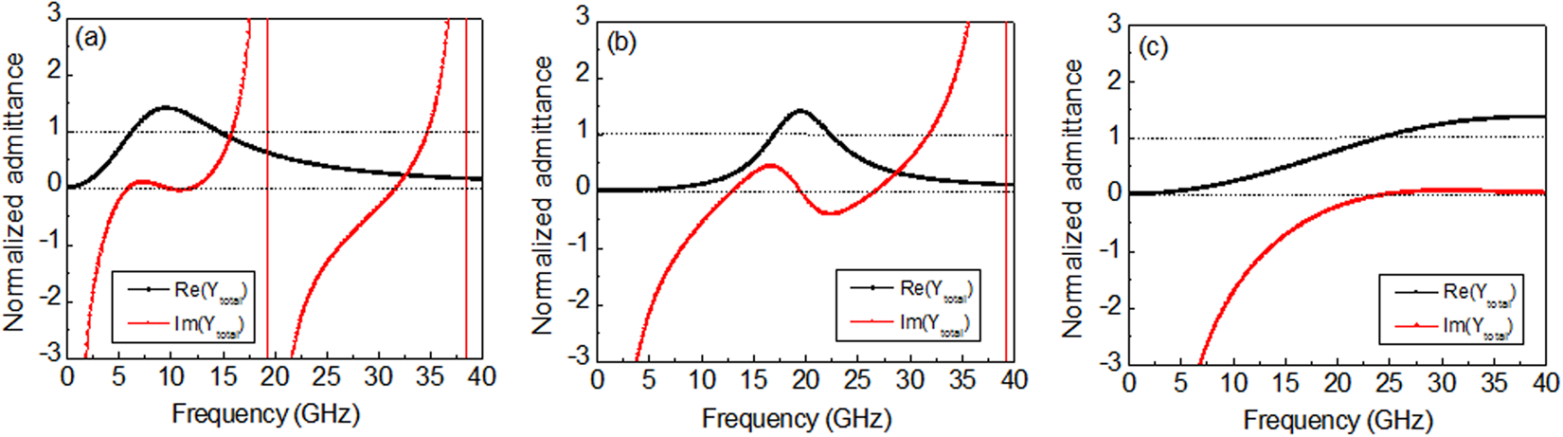
Normalized total admittance (Y_total_/Y_0_) of the grounded air substrate with FSSs: (**a**) SL-FSS (*R*_*s*_ = 20 Ω/sq, *t* = 7.8 mm), (**b**) C-FSS (*R*_*s*_ = 10 Ω/sq, *t* = 3.8 mm), and (**c**) SP-FSS (*R*_*s*_ = 100 Ω/sq, *t* = 2.0 mm).

Figure 3 depicts the normalized total admittance (Y_total_/Y_0_ = Re(Y_total_) + jIm(Y_total_)) of the grounded substrate with the FSS on the top surface. By combining the reverse pattern of the two admittances (i.e., the FSS and the grounded substrate), admittance matching can be derived over the wide frequency range with a controlled FSS resistance matched to the free-space impedance. Combining the SL-FSS with *R*_*s*_ = 20 Ω/sq with a grounded air substrate with a $\lambda /4$ thickness (*t* = 7.8 mm), the imaginary part of the total admittance flattens to zero (Im(Y_total_) ≈ 0) in a frequency range of 6–13 GHz, and the real part equals to the free-space admittance (Re(Y_total_) = 1) at two separated frequencies apart (6.4 GHz, 14.5 GHz), as depicted in Fig. 3(a). Admittance matching over the frequency interval leads to a broadband absorber. A similar pattern of total admittance was found for C-FSS (*R*_*s*_ = 10 Ω/sq) with admittance matching around 20 GHz ($\lambda /4$ = 3.8 mm), as seen in Fig. 3(b). For SP-FSS with *R*_*s*_ = 100 Ω/sq combined with a thin grounded air substrate (*t* = 2.0 mm), the admittance matching condition can be satisfied at high frequencies above 25 GHz, as depicted in Fig. 3(c), resulting in a broad-band absorber at high frequencies.

**Figure 4:**
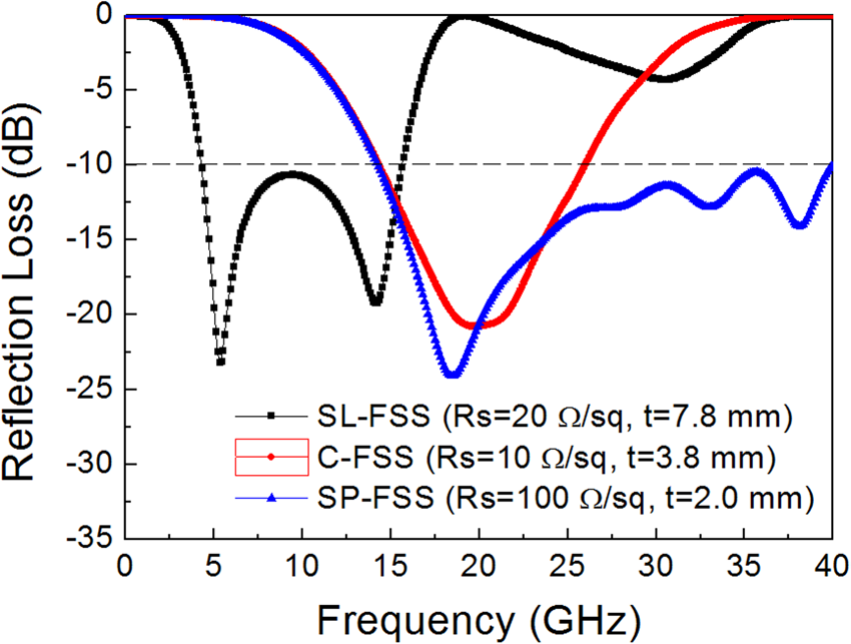
Simulation results of the reflection loss determined for the single-layer FSS absorber with a spacer thickness (*t*) and surface resistance (*R*_*s*_) indicated inside the figure.

Figure 4 presents the reflection loss for the single-layer FSS absorbers designed with an optimized admittance matching combination of FSS and substrate. For the SL-FSS (*R*_*s*_ = 20 Ω/sq) on a grounded air substrate (*t* = 7.8 mm), a broad bandwidth is predicted with a reflection loss lower than −10 dB in the frequency range of 4.3–15.6 GHz. For the C-FSS (*R*_*s*_ = 10 Ω/sq) on a grounded air substrate (*t* = 3.8 mm), the absorption frequency band is moved to the middle frequency range (14.4–26.0 GHz) with a reflection loss lower than −10 dB. For the SP-FSS (*R*_*s*_ = 100 Ω/sq, *t* = 2.0 mm), a broad bandwidth at high frequencies is predicted with a reflection loss lower than −10 dB in the frequency range above 14.3 GHz.

### Double-layer FSS Absorber Design

**Figure 5:**
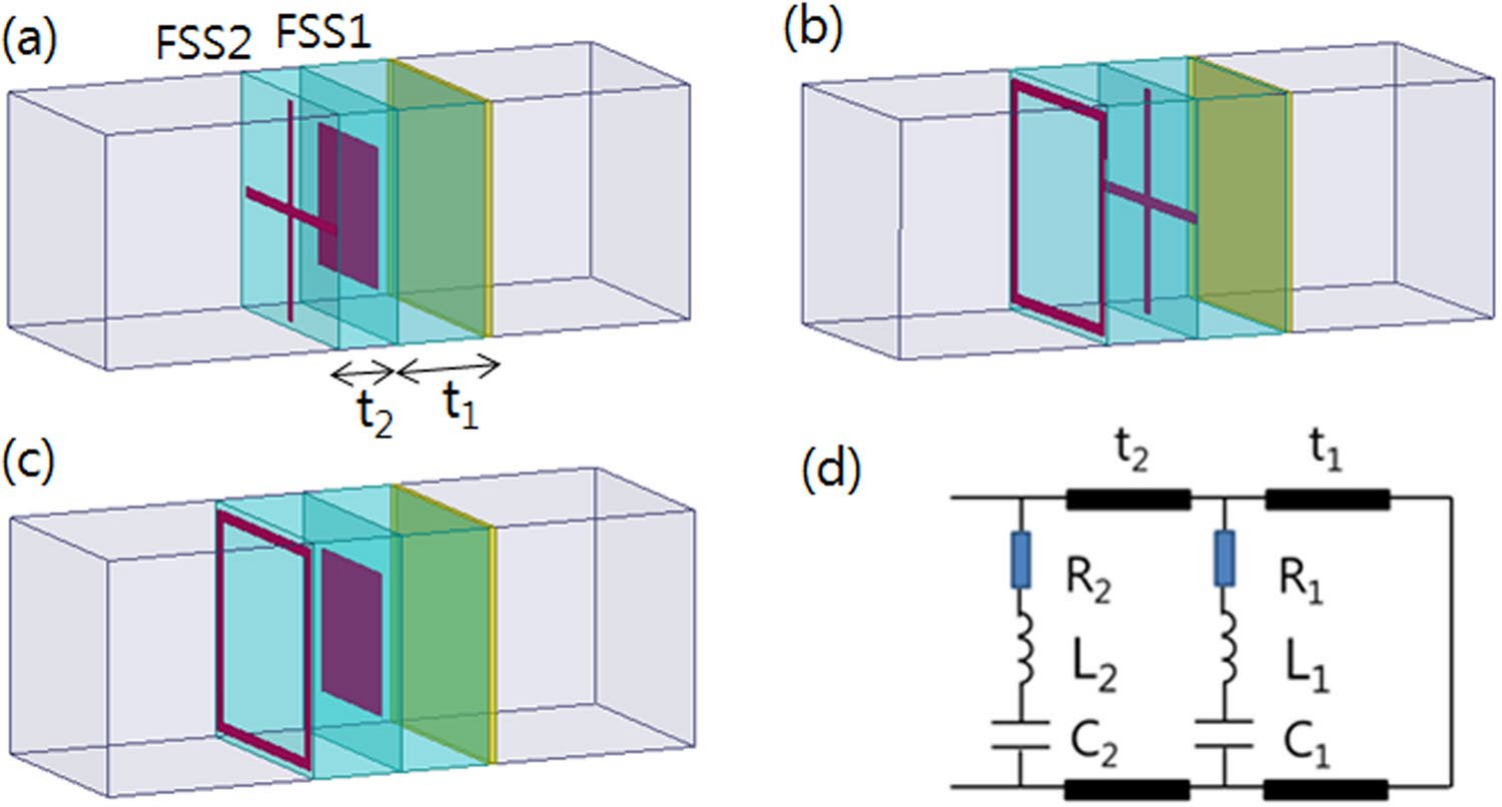
Schematic of the double-layered FSS absorbers with a combination of FSS1/FSS2: (**a**) square patch/cross, (**b**) cross/square loop, (**c**) square patch/square loop, and (**d**) equivalent circuit.

We designed a planar electromagnetic absorber with a wide bandwidth with a combination of the two resistive FSS layers, as illustrated in Fig. 5; the operation of the FSS absorber is explained using the equivalent circuit presented in Fig. 5(d). For the double-layer FSS absorbers, FSS1 was placed on the grounded substrate with a thickness of *t*_1_ and FSS2 was placed on the second substrate with a thickness of *t*_2_. The substrate was assumed to be air in order to use the *L* and *C* values of the FSS determined previously.

The first principle for the design of double-layer absorber is that the lower-frequency resonating FSS should be placed at the position of FSS2 because of the larger substrate thickness of a short-circuit resonance. For the FSS1 positioned at a shorter distance from the ground plane, the higher-frequency resonating FSS with lower inductance and capacitance should be selected. In the double-layer FSS absorber, however, multiple reflections occur at both FSS1 and FSS2 especially at a band in which two absorption peaks of each single-layer absorber overlap, which requires the modification of the design parameters of FSS resistance and spacer thickness. Table 4 presents the optimized design parameters for the double-layered FSS absorbers (surface resistance *R*_s_ and spacer thickness *t*) with the widest bandwidth.

**Table 4:** Optimized design parameters for double-layered absorbers with different FSS1/FSS2 combinations depicting the widest bandwidth.

FSS1/FSS2	*R*_s1_/*R*_s2_ [Ω/sq]	*L*_1_/*L*_2_ [nH]	*C*_1_/*C*_2_ [fF]	*t*_1_/*t*_2_ [mm]
Patch/Cross	100/10	0.74/6.23	26.56/10.62	2.0/1.0
Cross/Square loop	10/25	6.23/2.96	10.62/83.04	3.0/3.0
Patch/Square loop	100/20	0.74/2.96	26.56/83.04	2.0/3.5

**Figure 6:**
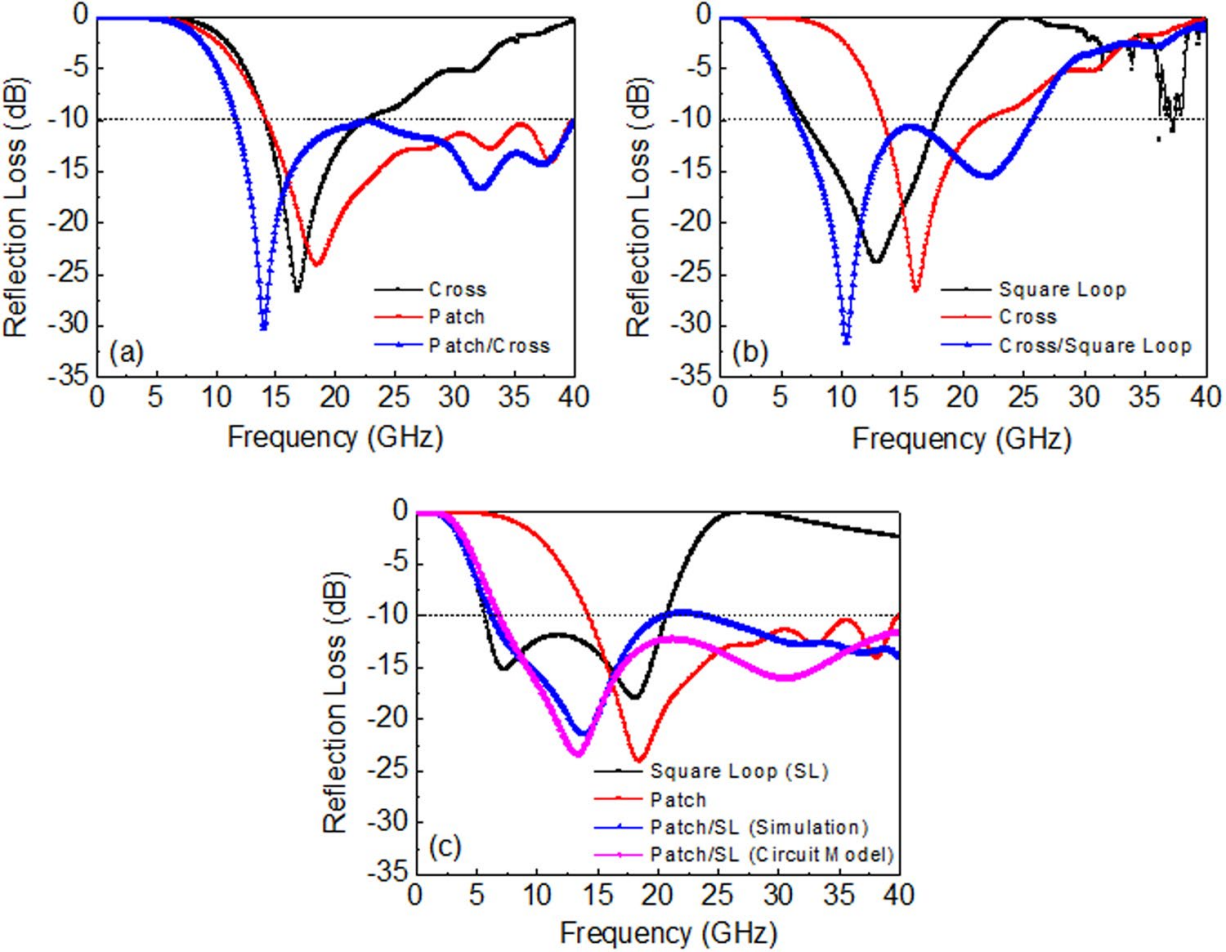
Simulation and calculated results of the reflection loss for the double layered absorbers with different FSS1/FSS2 combinations of optimum design parameters presented in Table 3: (**a**) patch/cross, (**b**) cross/square loop, and (**c**) patch/square loop.

Figure 6 presents the simulation results of the broad bandwidth characteristics of the double-layer FSS absorbers. For the first combination, the square patch/cross (FSS1/FSS2), the 10 dB absorption bandwidth was not greatly improved because the two absorption bands of each single-layer absorber overlapped, as seen Fig. 6(a). For the second FSS1/FSS2 combination, the cross/square loop, it is evident that the bandwidth broadened to a large value, which is approximately the sum of the bandwidth of each single-layer absorber of the cross (FSS1) and the square loop (FSS2), as seen Fig. 6(b). The widest bandwidth was predicted through the combination of patch/square loop for FSS1/FSS2. At the values *R*_s1_ = 100 Ω/sq, *R*_s2_ = 20 Ω/sq, *t*_1_ = 2.0 mm, and *t*_2_ = 3.5 mm, the reflection loss was decreased to less than −10 dB in the frequency range of 6.3–40.0 GHz, as depicted in Fig. 6(c). The reflection loss calculated from the circuit model of the transmission line is also provided in Fig. 6(c), which exhibits values for the absorption bandwidth and the reflection loss almost identical to those determined using the HFSS simulation. It is evident that the bandwidth of the double–layer absorber was maximized through the combination the two FSSs whose resonance frequency was sufficiently separated that their original absorption bands did not much overlap.

As depicted in Fig. 6, the low-frequency absorption peak of the double-layer absorber almost coincides with that of the single FSS2 absorber. At high frequencies, we observe a nearly identical reflection loss as that of the single FSS1 absorber. Basically, the absorption mechanism of the double-layer FSS absorber follows the principle of the high-impedance surface with the optimal resistive FSS of the single-layer absorber at the side region of absorption frequency band. In the middle frequency region, however, multiple reflections occur at both FSS1 and FSS2, which raises the reflection loss, especially at a band in which two absorption peaks of each single-layer absorber overlap. This required the modification of the design parameters.

**Figure 7:**
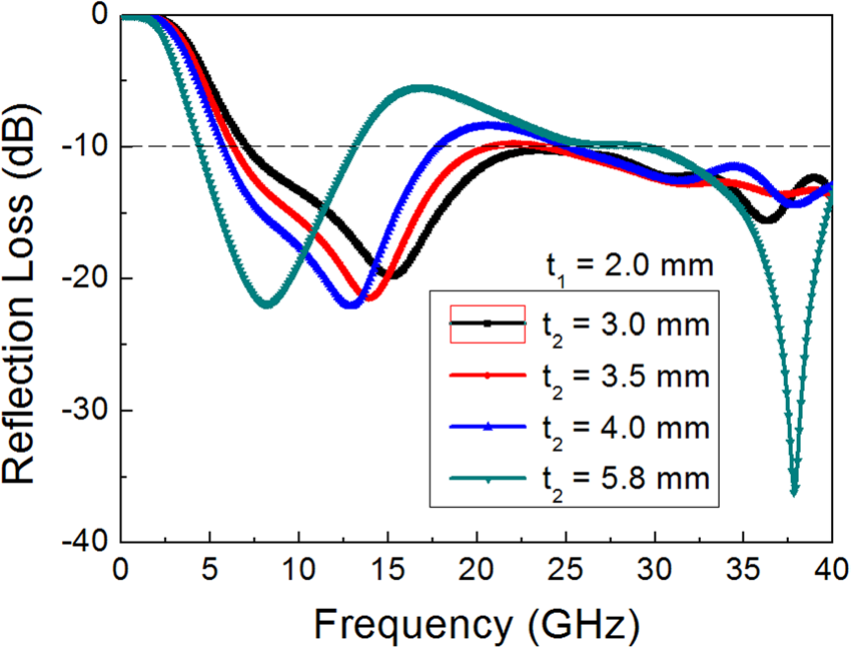
Simulation results of the reflection loss for the double layered absorbers with a patch/square loop (FSS1/FSS2) combination with reducing the second substrate thickness (*t*_2_) at a constant *t*_1_ = 2.0 mm.

For the double-layer absorber, the most critical design parameter for the widest bandwidth is the second spacer thickness (*t*_2_). The other parameters of surface resistances are the same as those of the constituent FSSs of single-layer absorber, as shown in Table 2. Figure 7 depicts the enhancement of absorption for the double-layered (patch/square loop) absorber with reducing the thickness of the second substrate from *t*_2_ = 5.8 mm to *t*_2_ = 3.0 mm, at a constant *t*_1_ = 2.0 mm. In the case of *t*_2_ = 5.8 mm, the reflection loss is higher than −10 dB in the middle frequency region, due to the multiple reflections at both FSS1 (patch) and FSS2 (square loop). Reduction of *t*_2_ lowers the reflection loss in that middle frequency region, due to the readjusted coupling between FSS1 and FSS2 toward to a new admittance matching. The enlargement of inductive frequency region with decreasing the second substrate thickness has an effect to compensate the existing capacitive circuit behavior of FSS1 (patch), which makes it possible to approach an admittance matching in the middle frequency region of strong coupling. At the lowest operation frequency band where the coupling is very small, however, wave reflection from FSS2 increases due to the deviation from the resonance thickness ($\lambda /4$ = 7.8 mm), resulting in a bandwidth reduction. The optimum value for the second substrate thickness with the maximum bandwidth is thus predicted to be *t*_2_ = 3.5 mm.

Rozanov proved that for a given absorption frequency response, the total thickness of the absorber (*t*) cannot be less than the theoretical limit^1^, as follows:

4 $${\rm{t}}\ge \frac{1}{2{\pi }^{2}}|{\int }_{0}^{\infty }\,{ln}|{\rm{\Gamma }}(\lambda )|d\lambda |,$$

where Γ is the reflection coefficient in dB and *λ* is the wavelength. This minimum possible thickness can be used as an evaluation tool to determine the efficiency of the design method. Using the absorption bandwidth result (Γ = −10 dB in the range of 6.3–40 GHz), the theoretical limit of the total thickness was estimated to be *t* = 4.7 mm, which is dominantly determined by the lowest operation frequency. The total thickness of the present work was 5.5 mm, which is close to the minimum possible thickness.

### Experimental Verification and Angular Stability

**Figure 8:**
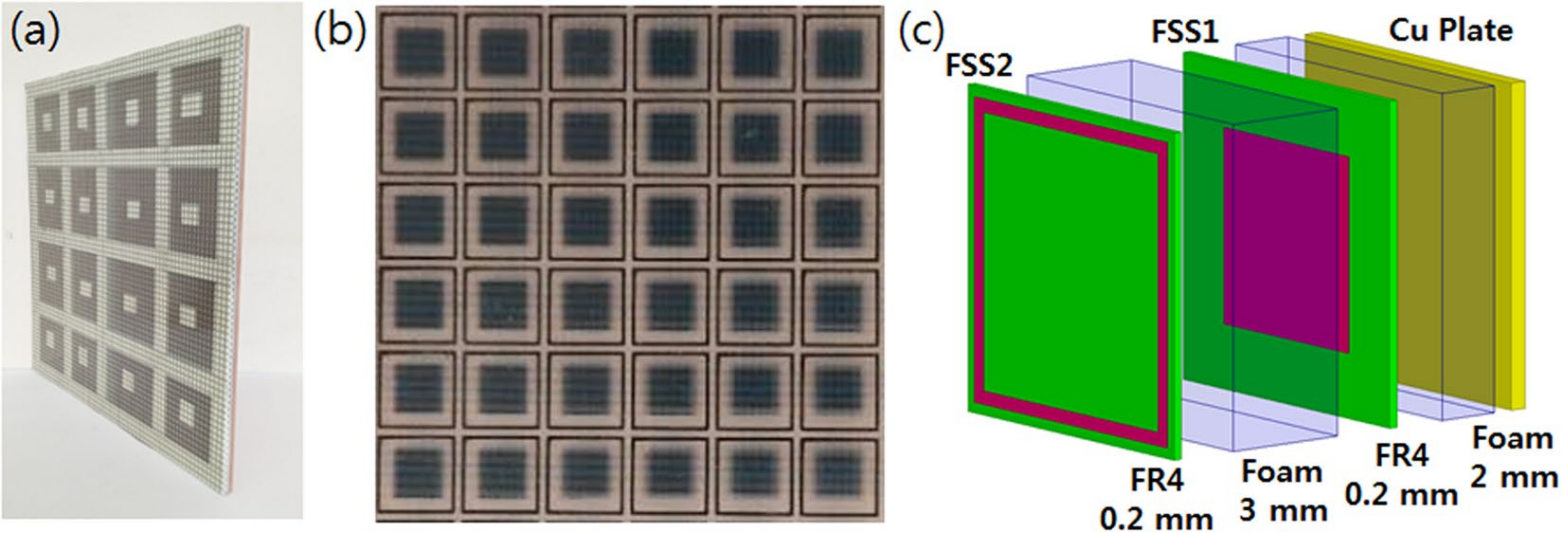
The test sample of a double-layer FSS absorber (size = 50 cm × 50 cm) for the measurement of reflection loss: (**a**) side view, (**b**) top view of printed FSS2/FSS1 patterns with a unit cell periodicity of 7.5 mm, and (**c**) a schematic layer structure.

The FSS1 (square patch) and FSS2 (square loop) with geometries and surface resistances close to the optimum values (Table 3) were fabricated by a screen printing method. Resistive ink material of carbon black paste, FR-4 plate used as the printing substrate, and the space material of open-cell polyethylene form were the same as those used in the previous study^28^. The surface resistance measured by a 4-probe technique was 100 Ω/sq for FSS1 (square patch) and 20 Ω/sq for FSS2 (square loop). The thickness of space material was controlled to be *t*_1_ = 2.0 mm and *t*_2_ = 3.0 mm. The back side was terminated by a copper plate. Figure 8 shows the test sample with a size of 50 cm × 50 cm with printed FSS pattern and its schematic layer structure.

**Figure 9:**
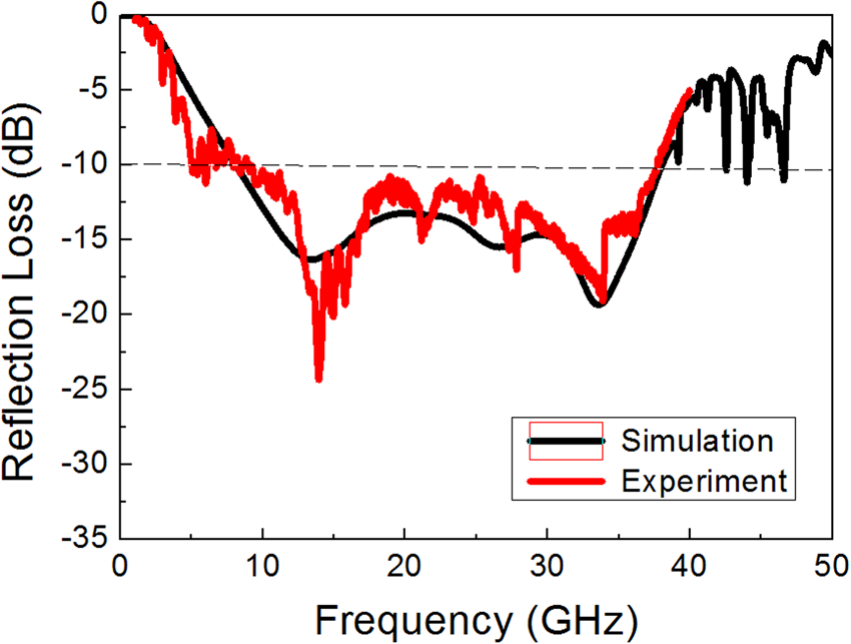
Measured reflection loss of the double-layer absorber with the structure presented in Fig. 8(c), Simulation result for the same double-layer structure and surface resistances as the test sample is also given for comparison.

The power reflection loss was measured by the free space measurement system. Figure 9 presents the measurement results of the reflection loss, depicting a 10 dB absorption bandwidth of 8–38 GHz, which is in good agreement with the simulation results for the same multilayer structure and surface resistances of the test sample. Compared with the air substrate, the bandwidth was slightly decreased due to the insertion of high-permittivity materials (FR-4) and a slight change in surface resistance and spacer thickness. Nevertheless, the measurement result strongly verified the validity of the proposed design method.

**Figure 10:**
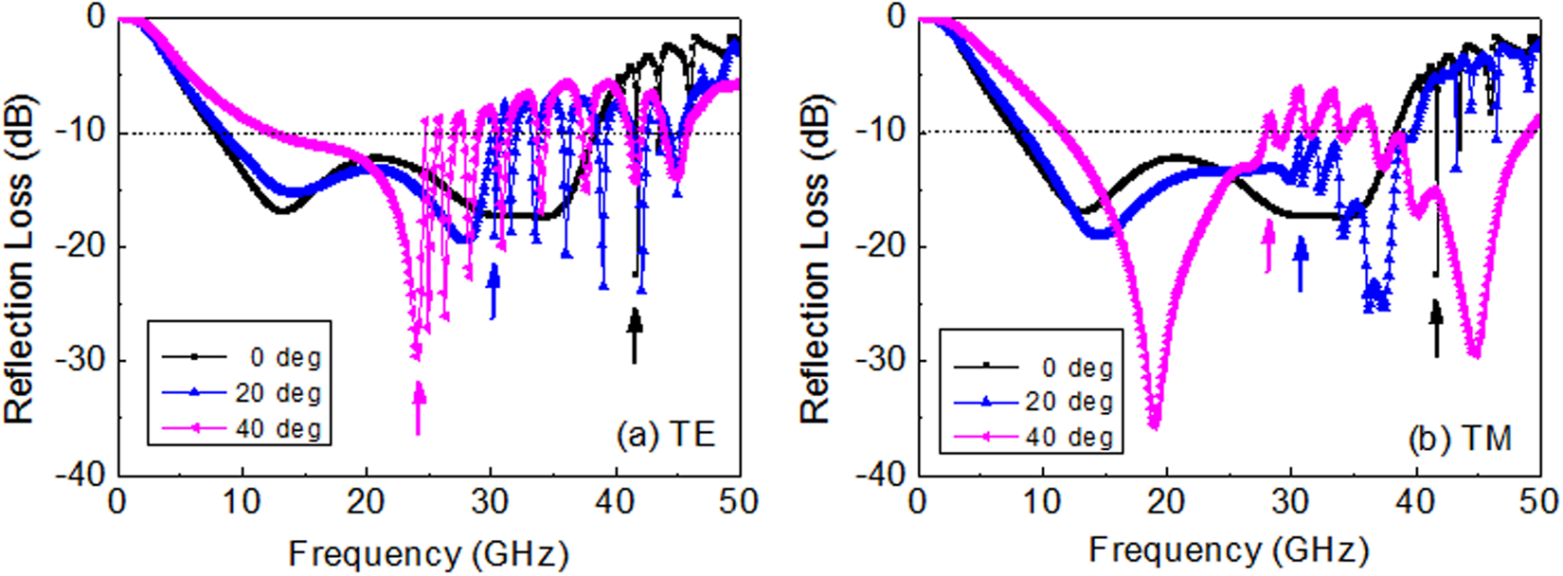
Simulation results of reflection loss of the double-layer FSS absorber with an increasing incidence angle for (**a**) TE and (**b**) TM polarization. The arrows inside the figures indicate the critical frequency at which the grating lobe appears.

The angular stability of the proposed wide bandwidth absorber was investigated over wide incidence angles for both TE and TM polarization. The grating lobes or high frequency harmonics were observed in the high frequency region above which the unit cell period was larger than wavelength (p ≥ λ)^6,32^. In the periodic array structure, it is possible that the array will have strong radiation in other directions from specular reflection. These unintended beams of radiation are known as grating lobes, which appear as many small peaks of reflection above the critical frequency. They occur in uniformly spaced arrays when the element separation or incidence angle is too large. For the normal incidence, the critical frequency was about 40 GHz for which the wavelength was equal to the FSS periodicity (p = 7.5 mm), as depicted in Fig. 9. As the incidence angle (θ) increased, the grating lobe occurred at a lower frequency for both TE and TM polarization, as indicated by arrows in Fig. 10. For θ = 40°, the critical frequency decreased to as low as 24 GHz (for TE) and 27 GHz (for TM). The unit cell periodicity must be controlled to less than a critical value in the periodic array structure, particularly for large incidence angles, which is approximately given by the equation p ≤ λ/(1 + sinθ)^33^.

## Discussions

A thin, ultra-wide bandwidth microwave absorber has been demonstrated utilizing two types of FSSs with different resonating frequencies. The circuit parameters, inductance and capacitance, of three types of FSS (square loop, cross, square patch) were determined using an equivalent circuit and a strip wire conductor model. The square loop FSS depicted a low frequency of resonance (10 GHz) due to its high values of inductance and capacitance. On the other hand, the square patch of small inductance revealed a high resonating frequency (36 GHz). By combining the two FSSs, we designed an ultra-wide absorption bandwidth (6.3–40.0 GHz for −10 dB reflection loss) at a small total thickness of 5.5 mm for an air substrate, which is close to the theoretical limit. We could state the possible mechanism based on the coupling behavior between the double-layer FSSs. This study has novelty and significance over the existing works from standpoints of enlarged bandwidth (fractional bandwidth 145%) with a comparable thickness (0.11λ_L_), and simple structure (only two FSS layers) of easy fabrication. The free space measurement result with a test sample prepared by the screen printing method was in good agreement with the simulation result and strongly verified the validity of proposed design method. For these periodic array structures, however, the grating lobes or high frequency harmonics were observed in the high frequency region above which the unit cell periodicity is larger than the wavelength, and as the incidence angle increased, the grating lobe occurred at a lower frequency for both TE and TM polarization. The unit cell periodicity must be controlled in the periodic array structure, particularly for large oblique incidence angles.

## Methods

The proposed FSS absorbers were simulated and optimized in ANSYS-HFSS using master-slave periodic boundary conditions and two Floquet ports to illuminate both sides of the FSS absorbers. To verify the simulation results, a 66 × 66 unit cell array was fabricated and measured in an anechoic chamber. The FSSs with geometries and surface resistances close to the optimum values (Table 3) were fabricated by a screen printing method. Carbon black paste was chosen as the resistive ink, and a commercially available FR-4 plate (with permittivity of *ε*_r_ = 4.05 + j0.017 and 0.2 mm thickness) was used as the printing substrate. The surface resistance of FSS strips was measured by a 4-probe technique. The space material was an open-cell polyethylene form with a permittivity of *ε*_r_ = 1.05 + j0.02. Measurements were carried out using an Agilent vector network analyzer and two horn antennas for three different frequency bands (1–18 GHz, 18–30 GHz, 26.5–40 GHz) to obtain the reflection coefficients. After calibration with a perfectly reflecting copper plate, measurements of power reflection were made for the test samples.
